# Combination of the PARPi and ARSi in advanced castration resistant prostate cancer: a review of the recent phase III trials

**DOI:** 10.37349/etat.2024.00260

**Published:** 2024-08-02

**Authors:** Martina Panebianco, Vittore Cereda, Mario Rosario D’Andrea

**Affiliations:** NGO Praeventio, Estonia; Medical Oncology of ASL Roma 4 Hospital, 00053 Civitavecchia, Italy

**Keywords:** Prostate cancer, PARPi, ARSi, combination strategies, castration resistant, BRCA

## Abstract

Tumors with an impaired ability to repair DNA double-strand breaks by homologous recombination, including those with alterations in breast cancer 1 and 2 (*BRCA1* and *BRCA2*) genes, are very sensitive to blocking DNA single-strand repair by inhibition of the poly (ADP-ribose) polymerase (PARP) enzyme. This provides the basis for a synthetic deadly strategy in the treatment of different types of cancer, such as prostate cancer (PCa). The phase 3 PROfound study was the first to lead to olaparib approval in patients with metastatic castration resistant PCa (mCRPC) and *BRCA* genes mutations. In recent years, the benefit of combination therapy consisted of a PARP inhibitor (PARPi) plus an androgen receptor signalling inhibitor (ARSi), was evaluated as first-line treatment of mCRPC, regardless of the mutational state of genes, participating in the homologous recombination repair (HRR). This review explores the role of PARPi in PCa and analyses the data of latest clinical trials exploring the PARPi—ARSi combinations, and how these results could change our clinical practice.

## Introduction

### Prostate cancer and *BRCA* mutation: incidence and prognosis

Cancer cells may have defects in DNA repair mechanisms leading to genomic instability and promoting oncogenesis. It has been shown that different tumors with an inability to fix DNA double-strand breaks (DSB) by homologous recombination repair (HRR), such as those with alterations in the breast cancer 1 and 2 (*BRCA1* and *BRCA2)* genes, are very responsive to an emerging therapeutic approach, represented by the inhibition of the poly (ADP-ribose) polymerase (PARP) enzyme. The introduction of PARP inhibitor (PARPi) in prostate cancer (PCa) is a milestone and provides a pathway to hope in fighting this disease [1–3].

The incidence of germline mutations in DNA damage response *(DDR)* genes among men with metastatic PCa fluctuates between 11% and 33%. It has been demonstrated that about 10% of patients with PCa has a mutation in *BRCA1* and/or *2* genes [4, 5]. However, the rate of *BRCA* mutated patients with advanced castration resistant PCa (CRPC) is variable between different clinical studies, depending on the type of genetic panel used. A study on 692 patients with advanced PCa showed that 84 patients (11.8%) had a mutation in homologous DNA repair genes, of which 5.2% had a *BRCA2* mutation [6]. Another very interesting research identified mutational signatures through the analysis of the whole-genome and -transcriptome sequencing of 101 PCa metastases. Inactivation of *CDK12*, *TP53*, and *BRCA2* affects distinct classes of structural variants. Nevertheless, from this investigation, we do not have yet clear data on the functional consequences of different mutations and the potential predictive role of the identified signatures [7]. Patients with metastatic CRPC (mCRPC) and *BRCA* defects, especially *BRCA2*, have significantly worse progression-free survival (PFS; 3.7 months vs. 9.8 months), overall survival (OS; 18.9 months vs. 33.9 months) and PSA response rates to androgen receptor (AR) pathway inhibitors (32% vs. 60%, *P* = 0.02) than *BRCA* wild type (*BRCA* WT) patients [8]. Since it has become clear that PCa is associated with a frequent incidence of mutations in DNA repair genes, PARPis have also been included in the therapeutic armamentarium of this pathology.

### Repair mechanisms of DNA damage and tumor synthetic lethality

There are several repair mechanisms available in our cells. The repair pathways for base excision repair (BER), nucleotide excision or incorrect base pairing intervene when just one DNA strand is damaged, and the intact complementary strand can be used as a template. Instead, the DSBs are repaired by HRR or non-homologous end joining (NHEJ) pathways [9]. Microhomology-mediated end joining (MMEJ) is an alternative DSB repair pathway to NHEJ [10]. The MMEJ seems to be a crucial DSB repair mechanism for HRR- and NHEJ-defective tumors [11]. The DSB damage is more complex to solve than single strand breaks (SSBs) because the complementary DNA strand is not ready for use as a model [11].

PARP-1 is an enzyme that produces large and branched chains of poly ADP-ribose. PARP-1, which is abundantly expressed in the cell nucleus, detects, and binds DNA discontinuities, resulting in activation of catalytic activity, causing poly-ADP ribosylation of PARP-1 itself, as well as of other acceptor proteins, including histones. This could be the signal for the recruitment of other components of DNA repair pathways [12]. As previously hinted, within the cell, two main DSB repair pathways are actionable: NHEJ and HRR. HRR can be further subdivided into gene conversion (GC) and single strand annealing (SSA) [13]. Both GC and SSA depend on sequence homology for repair, as opposed to NHEJ. In fact, while NHEJ repair is guided by short homologous DNA sequences (called micro-homologies), present in single-stranded overhangs on the ends of DSB to fix the damage, GC relies on a homologous sequence, usually the sister chromatid, which is used as a true model for synthesizing DNA in correspondence with the DSB, resulting in a precise repair of DNA break [14]. When the NHEJ pathway is inactivated, DSBs can be repaired by MMEJ, which uses short microhomologies on either side of the break, which are then aligned to guide repair. This mechanism contrasts with classical NHEJ, which uses microhomologies, already exposed in single-stranded overhangs on the DSB ends [10].

The *BRCA1* and *BRCA2* were classified as tumor suppressor genes. They encode large proteins that function in multiple cellular pathways, such as transcription, regulation of the cell cycle, and maintaining genome integrity. BRCA1 has multiple functions, while it has been demonstrated that BRCA2 has an essential role in DNA repair mechanisms, through the assembly of the RAD51 recombinase. Indeed, BRCA2 is fundamental for the localization of RAD51 at DNA damage sites, where RAD51 forms the nucleoprotein filament needed for homologous recombination [15]. Interestingly, several studies have documented that loss of wild type BRCA-2 increases, at least in part, error-prone repair mechanisms, such as those regulated by the SSA pathway. Thus, *BRCA2* mutation may result in activating non-conservative HRR, leading to greater chromosomal instability [16].

Inhibition of PARP-1 affects just SSB repair but it has been shown that multiple SSBs produces the disruption of the DNA replication fork leading to DSB. When PARP is inhibited by a pharmaceutical agent in the presence of a deficiency in the function of both *BRCA1* and *BRCA2* genes, thus inactivating GC mechanisms, DNA damages could be just repaired by non-homologous repair strategies. Alternative error-prone DSB repair mechanisms, such as SSA or NHEJ, may have a major role in this context, leading to the subsequent large number of chromatin aberrations and ultimately cell death. Thus, PARPis are selectively fatal to cells with non-functional BRCA1 or BRCA2 [17]. Several preclinical studies have demonstrated that the lack of other genes, besides *BRCA1* and *BRCA2*, involved in HRR mechanisms, may be predictive for the efficacious use of PARPi in cancer cells [18, 19]. About 20% of advanced PCa have a mutation in *BRCA1*, *BRCA2,* or *ATM* genes. In line with the phenomenon of synthetic lethality, the data demonstrated a significant clinical response using PARPis in CRPC patients with *ATM*, *BRCA2,* or *BRCA1* mutation [20]. Some authors showed that even the deficiency of other genes, including *RAD51*, *RAD54*, *DSS1*, *RPA1*, *NBS1*, *ATR*, *ATM*, *CHK1*, *CHK2*, *FANCD2*, *FANCA*, or *FANCC* induce PARPi sensitivity [18]. Moreover, other data suggest that PCa cells with loss of *MMS22L* are equally sensitive to PARP inhibition [21]. Recently, in this regard, some researchers have aimed to further personalize treatment with PARPi. For example, it was identified a group of patients carrying loss of both alleles of the *CDK12*, that is potentially targetable with immune checkpoint inhibitors [22] and not sensitive to PARPi [23]. In fact, the *CDK12* seemed to regulate genes involved in the DDR and thus controlled genomic stability and the loss of the *CDK12* was described as a resistant mechanism to PARPi in ovarian cancer [22].

At the same time, new clinical trials of PARPi in mCRPC evaluated the efficacy of the combination of PARPi with an AR signalling inhibitor (ARSi), including a population not selected for *HRR* gene mutation [24–26].

### The synergistic activity of the PARPi and ARSi combination strategy

The biological rationale of the combination is based on a synergistic antitumor activity of the ARSi and the PARPi [27]. Indeed, as shown by Asim et al. [28], the inhibition of the AR pathway contributes to the down-regulation of *HRR* gene expression in PCa. It is well established that RAD51 protein expression is significantly increased in PCa tissues, as compared with benign prostate samples. Authors demonstrated that in an AR-knockdown cell model the expression of several proteins, such as meiotic recombination 11 (MRE11), implicated in HRR repair, is reduced [28]. In particular, MRE11 is a part of the MRE11/RAD50/NBS1 (MRN) complex, a sensor of DSBs, and physically localizes to sites of damage rapidly after the insult and it is fundamental in HRR [29]. These results were validated in a prospective cohort of PCa patients receiving neo-adjuvant leuprolide. Tissue samples were collected before leuprolide treatment and 8 weeks after the end of anti-hormonal therapy. It was observed that patients, being subjected to Androgen Deprivation Therapy (ADT) before radiation, had PCa tissues with a lower percentage of RAD51-positive cells, as compared with those treated with radiation alone [28].

**Figure 1:**
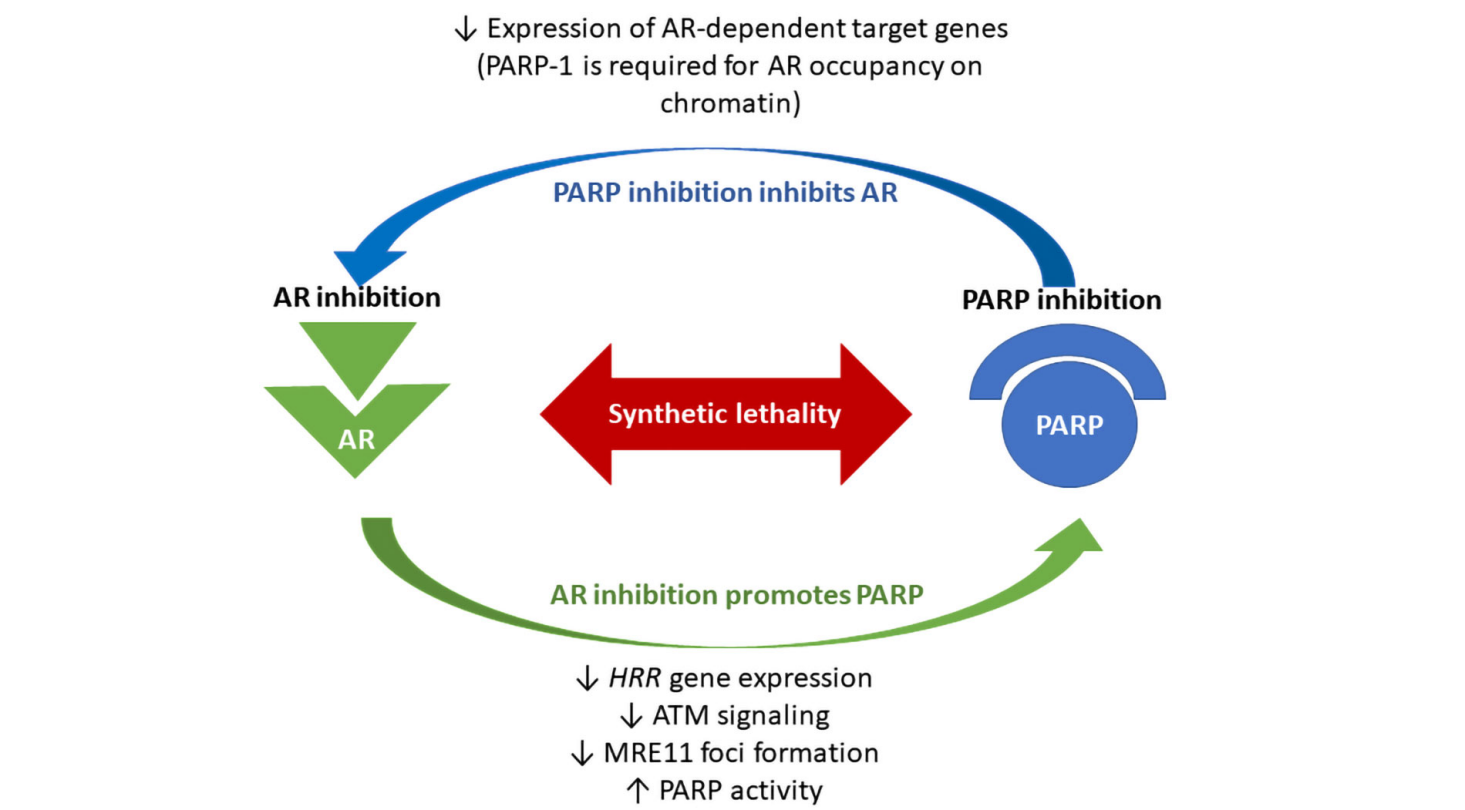
Biological synergism between poly (ADP-ribose) polymerase (PARP) and androgen receptor (AR) inhibitors. The loss of the AR leads to downregulated homologous recombination repair (*HRR*) gene expression in prostate cancer (PCa). Androgen Deprivation Therapy (ADT) can functionally impair HRR, through inhibition of ataxia telangiectasia mutated ATM signaling and inhibition of meiotic recombination 11 (MRE11) foci formation. It has been shown that enzalutamide may suppress the expression of *HRR* genes in castration resistant PCa (CRPC) cells, creating HRR deficiency and BRCAness status. At the same time, PARP is required for survival in the absence of HRR, and PARP activity is increased in PCa tissue, following ADT. Therefore, it has been demonstrated that PARP exerts a dual role: regulating DNA repair mechanisms and supporting AR activity and resistance to genotoxic insult in PCa. PARP inhibition leads to a significant down-regulation of AR-target gene expression. All this evidence explains the synthetic lethality between ADT and PARPi

PARP is essential for cell survival in the absence of HRR mechanism, and it was demonstrated that it is up-regulated in PCa samples, following the depletion of *HRR* genes [30]. At the same time, a study by Schiewer et al. [31] revealed that expression of AR-dependent target genes was decreased in a cellular model treated with a PARPi called ABT888, since PARP-1 itself regulates AR function through its association with chromatin. These data were confirmed also in vivo models with a significant down-regulation of AR-target genes expression in mice treated with ABT888, as well as with olaparib. Authors also documented that the combination of castration and inhibition of PARP lead to a significant prolongation (about 50%) of tumor doubling time, as compared with that observed in models treated with castration alone. Due to these data, it seems that agents targeting PARP-1 not only concur with castration to an in vivo decrease tumor growth but also limit the progression from hormone-sensitive PCa to lethal CRPC. Therefore, it has been shown that PARP exerts a dual role: it repairs DNA alterations and it regulates AR activity, making PCa cells resistant to mutagenic injuries [31]. Concurrent AR inhibition by ADT weakens HRR mechanisms and this should activate PARP function, which, if it is at the same time blocked, may not fix DNA damages, resulting in cancer cell death [31]. An interesting report demonstrated that also enzalutamide, an AR inhibitor, may down-regulate *HRR* genes, leading to a BRCAness status [32]. All this evidence explains the synergic effect between ADT and PARPi (Figure 1).

In this review we will explore the recent clinical role of PARPi in PCa, discuss the data of the latest clinical trials, that have evaluated the combination of the PARPi with the ARSi in an earlier line of treatment and regardless mutational status, and we’ll wonder if these results will change our clinical practice.

## New strategies in mCRPC: the new era of the PARPi

### The earlier use of PARPi in treatment strategy of PCa

In the last few years, several PARPis have been developed. Different phase II and III clinical trials have investigated the efficacy and safety of olaparib, niraparib, rucaparib, talazoparib and veliparib with variable results in metastatic PCa patients, following disease progression after several treatment lines [3, 33]. Since patients will eventually become resistant to PARPi, a current field of clinical research is focusing on understanding the clinical benefit of PARPi in an earlier stage of disease [33]. Within this setting, PARPis are evaluated alone or in combination with different other agents (ARSi, immunotherapy, radiotherapy, chemotherapy, targeted therapy) [3]. Furthermore, a new generation of selective PARP-1 inhibitors (such as AZD5305, also called saruparib) is being developed, in order to obtain more clinical efficacy and reduce toxicity [34, 35].

Recently, a phase III trial, called TRITON3, showed the major efficacy of earlier use of a PARPi (rucaparib) in the sequence treatment of *BRCA* mutated mCRPC patients [36]. Rucaparib is a PARPi that demonstrated significant anti-tumor activity in the TRITON2 phase II study in patients with *BRCA* mutated mCRPC, pre-treated with an ARSi and/or chemotherapy based on taxanes [37]. The TRITON3 study, discussed during 2023 ASCO GU cancer meeting, enrolled 405 patients with *mCRPC* and *BRCA* or *ATM* mutation, progressing to previous treatment with ARSi but not with chemotherapy in the castration resistance setting. The patients were randomized to rucaparib versus physician’s choice treatment (docetaxel or ARSi, enzalutamide or abiraterone). Patients, allocated in the control arm, were allowed to crossover to rucaparib upon disease progression (the treatment was received in 75% of cases). Rucaparib significantly increases radiological PFS (rPFS) in mutated *BRCA* tumours, reducing the risk of progression by 50% (rPFS: 11.2 months vs. 6.4 months; HR: 0.50; *P* < 0.001) and in the intention to treatment (ITT) population (HR: 0.61; *P* = 0.003). The advantage of rucaparib in rPFS was evident not only with respect to the ARSi control arm (rPFS: 11.2 months vs. 4 months; HR: 0.38; *P* < 0.001), which was a weaker control arm, since patients received another ARSi as first line treatment, but also as compared with the more effective docetaxel control arm (rPFS: 11.2 months vs. 8.3 months; HR: 0.53; *P* = 0.009). However, rucaparib benefit in rPFS was not demonstrated in the *ATM* mutated population (HR: 0.95; 95% CI: 0.59–1.52). Preliminary survival analysis failed to demonstrate an advantage of rucaparib in the mutated *BRCA* population over the control arm, but the data are still immature (54% of events in the *BRCA* mutated population). Moreover, about 3/4 of the patients in the control arm received rucaparib upon progression and this crossover could negatively affect the final survival data. With regard to toxicity profile, asthenia/fatigue was the most common treatment-related adverse event (TRAE) in all treatment arms, followed by nausea and anemia [36]. On May 2020 FDA granted accelerated approval for rucaparib for the treatment of *BRCA* mutated mCRPC patients that have progressed to ARSi and taxane-based chemotherapy.

A second PARPi approved in PCa was olaparib. In 2020, this agent demonstrated an interesting clinical efficacy in patients with mCRPC and with mutations in *BRCA1/2* genes (germline mutation and/or somatic mutation), progressing after chemotherapy [3, 38]. Subsequently, PROfound trial has been the first randomized phase III trial to show a clear benefit of olaparib, as opposed to an ARSi (enzalutamide or abiraterone) in patients with mCRPC, who had disease progression after a prior ARSi [39, 40]. In view of these data, the FDA approved olaparib for patients with HRR genes alterations, while EMA restricted the approval only to patients with *BRCA1* and *BRCA2* defects. On the basis of the most recent clinical trials, today olaparib is also indicated in combination with abiraterone in the first-line treatment of mCRPC, regardless of the mutational status [24].

### PARPi-ARSi combination strategy in phase III clinical trials

In mCRPC setting, due to the radical changes of the treatment of mCSPC patients, which inevitably lead to profound variations of the therapeutic algorithm of the castration-resistant state, research is currently focused on investigating the role of new approaches. In recent years, PARPi-ARSi combination strategy was evaluated as first-line treatment of mCRPC. Two studies investigated the role of the PARPi-ARSi combination in the mCRPC population not selected for HRR status in the first-line setting: PROpel and the TALAPRO-2 trials [24, 26].

The PROpel study was a multicenter, double-blinded, randomized (1:1) phase III trial with the purpose to assess the clinical benefit of a combination therapy, represented by abiraterone + olaparib (399 patients) versus abiraterone + placebo (397 patients) in mCRPC setting, independently from mutational status. Then, a post hoc analysis of tumor tissue and blood samples (ctDNA) tested several *HRR* genes (*ATM*, *BRCA1*, *BRCA2*, *BARD1*, *BRIP1*, *CDK12*, *CHEK1*, *CHEK2*, *FANCL*, *PALB2*, *RAD51B*, *RAD51C*, *RAD51D*, and *RAD54L*) and the patients were distinguished into three groups: HRR-mutated (HRRm, 28.4%), non-HRRm (69.3%) and HRRm unknown (2.3%). It has been shown that the two arms had different percentages of mutated *BRCA* patients (11.8% in the combination therapy arm with respect to 9.6% in the control arm). Overall, just 10% of the patients analyzed had mutations in *BRCA 1/2* genes. The primary endpoint of the study was investigator-assessed rPFS, with several secondary endpoints, comprising overall response rate (ORR), time to first subsequent treatment, time to second PFS or death, safety, and OS. At a primary median follow-up of nearly 19 months, the median rPFS was significantly prolonged in favor of the olaparib + abiraterone arm vs. abiraterone and placebo arm (24.8 months vs. 16.6 months; HR: 0.66; 95% CI: 0.54 –0.81; *P* = 0.001) [41]. In the subgroup analysis, the improvement of rPFS in patients receiving olaparib + abiraterone was confirmed, regardless of the previous castration sensitive therapy (ARSi or docetaxel) and in both the HRRm (28.8 months vs. 13.8 months; HR: 0.45; 95% CI: 0.31–0.65) and non-HRRm groups (27.6 months vs. 19.1 months; HR: 0.72; 95% CI: 0.56–0.93). With respect to patients with measurable disease at baseline (40.3%), the ORR was slightly increased in the combination therapy group vs. control group (58.4%, 94 of 161 patients vs. 48.1%, 77 of 160 patients; HR: 1.60; 95% CI: 1.02–2.53). The most common adverse events (AEs) in the experimental arm were anaemia (in particular, 15.1% vs. 3.3% of patients experienced a grade 3 or higher anaemia), fatigue/asthenia, and nausea [41]. Later, at a median follow-up analysis of 36.6 months (47.9% of events), rPFS was congruous with the primary analysis and in particular, it has been shown that in *BRCA* mutated subgroup, the rPFS was not reported (NR) for the combination treatment arm versus 23 months for the standard treatment population (HR: 0.29; 95% CI: 0.29–0.56) [24]. However, in the all-comers population OS was not statistically improved (HR: 0.81; 95% CI: 0.67–1.00; *P* = 0.0544) and the combination therapy did not demonstrate an OS advantage in non-mutated HRR patients (HR: 1.35; 95% CI: 0.70–1.39) [24]. At the same time, it was observed a significant benefit in the HRRm subgroup, especially in BRCA mutated patients, for whom the OS advantage of the combination became clear with an HR of 0.29 (95% CI: 0.14–0.56), compared to non-*BRCA* mutated tumours (HR: 0.91, 95% CI: 0.73–1.13) [24]. Nevertheless, also the authors specified that the *BRCA* mutated group was poorly represented (only 35 patients). In this secondary follow-up, the safety profile was confirmed with severe AEs observed in 55.8% of patients receiving olaparib + abiraterone vs. 43.2% receiving standard therapy. Anaemia was the most common grade 3–4 AE and occurred in 64 of 398 patients (16%) in the combination group and in 13 of 396 patients (3%) in the placebo plus abiraterone arm [24]. On December 2022 the European Commission (EC) approved the combination of olaparib plus abiraterone for the treatment of mCRPC patients not eligible for chemotherapy, because of clinical status and side effects [42].

The phase III TALAPRO-2 trial investigated the combination of talazoparib and enzalutamide in an unselected HRR population with mCRPC, as first line treatment [26]. Unlike the previous study, the HRR status, as well as previous treatments, was considered as stratification factor (deficient vs. non-deficient and docetaxel or abiraterone: yes vs. no, respectively). Of the 805 patients analyzed, only 20% of them were HRR-deficient, while 7% in the talazoparib group and 8% in the placebo group were *BRCA* mutated. At a median follow up of 24 months, the combination therapy demonstrated a reduction of the progression risk of 37% compared to the control arm. Median rPFS was not reached in the experimental arm vs. 21.9 months in the control arm; HR: 0.63; 95% CI: 0.51–0.78; *P* < 0.001, regardless of the *HRR* genes mutation. Anyway, this advantage was greater in the HRR-deficient group (HR: 0.46; 95% CI: 0.30–0.70; *P* < 0.001) as compared with patients having HRR-proficient or not known tumors (HR: 0.70; 95% CI: 0.54–0.89; *P* = 0.004) [26]. However, it was observed that the failure rate in tissue HRR molecular testing was about 28%. No advantage was observed in OS (HR: 0.89; *P* = 0.35) due to data immaturity (only 31% of events). With regard to the safety profile, the combination therapy was associated with a really significant increase in the rate of TRAEs of each degree (89.7% vs. 69.6%) and severe degree (19.6% vs. 3.0%). The major AE was anaemia (64.6% vs. 15.6% of control arm) and unfortunately 62% of patients receiving talazoparib + enzalutamide had a dose interruption of talazoparib as opposed with 21% of patients in the control arm. Moreover, in the experimental arm, there were one myelodysplastic syndrome and one acute myeloid leukaemia during the follow-up period [26].

The MAGNITUDE was a multicenter, double-blinded, randomized phase III study, assessing the efficacy of abiraterone acetate and prednisone (AAP) plus niraparib or placebo as first-line approach in mCRPC patients with and without mutation of *HRR* genes, containing *ATM*, *BRCA1*, *BRCA2*, *BRIP1*, *CDK12*, *CHEK2*, *FANCA*, *HDAC2*, or *PALB2*. Patients were prospectively assigned to HRR-positive or HRR-negative cohorts and subsequently randomized (1:1) to receive combination therapy or abiraterone alone. Inclusion criteria permitted up to four months of AAP prior to the enrolment, docetaxel in the castration sensitive setting or an ARSi in a non-mCRPC phase or for castration sensitive disease. Due to the absence of an advantage observed from the combination of niraparib and abiraterone in the HRR negative group, after the enrolment of nearly 200 patients, this cohort was closed for futility [25]. After 35 months of follow-up, rPFS of the combination treatment was significantly improved (19.5 months vs. 10.9 months; HR: 0.55; *P* = 0.007) in *BRCA* mutated subgroup. Nevertheless, the OS advantage of the combination was not demonstrated in the mutated *BRCA* population (HR: 0.88; 95% CI: 0.58–1.34; *P* = 0.055) [43]. At the final analysis, 225 patients with *BRCA* alterations were analyzed. 113 patients received niraparib plus abiraterone and 112 received abiraterone plus placebo [44]. The 3 years-update analysis, presented at ESMO congress in 2023, did not show a significant improvement in favor of the experimental arm in terms of OS (HR: 0.78; 95% CI: 0.554–1.12; *P* = 0.1828) [44]. Anyway, a pre-specified multivariable analysis, adjusting for baseline imbalances, showed a significant OS benefit favoring niraparib plus abiraterone (HR: 0.663; 95% CI: 0.464–0.947). In combination and standard treatment groups, treatment-related AEs occurred in 165 (77.8%) and 121 (57.3%) patients, respectively, and were congruent with the historical safety characteristics of the individual agents [44].

Table 1 summarizes the above-mentioned studies’ outcomes in patients with mCRPC.

**Table 1:** PARPi and survival outcomes in mCRPC: main trial presented at ASCO GU 2023

**Trial**	**Line setting**	**HRR status/BRCA status**	**Median FU (months)**	**rPFS** **(Months; experimental vs. standard)**	**OS** **(Months; experimental vs. standard)**
PROpel (Olaparib/placebo + abiraterone)	1L mCRPC	HRRm (30%)/non-HRRm BRCAm (10%)/overall population	36.6 m	ITT: 24.8 vs. 16.6; HR: 0.66, 95% CI: 0.54–0.81, *P* < 0.001 HRRm: NR vs. 13.9; HR: 0.50, 95% CI: 0.34–0.73 non-HRRm: 24.1 vs. 19.0; HR: 0.76, 95% CI: 0.60–0.97 BRCAm: NR vs. 8.4; HR: 0.23, 95% CI: 0.12–0.43 non-BRCAm: 24.1 vs. 19.0; HR: 0.76, 95% CI: 0.61–0.94	ITT: 42.1 vs. 34.7 maturity 47.9%; HR: 0.81, 95% CI: 0.67–1.00; *P* = 0.0544 HRRm: NR vs. 28.5; HR: 0.66, 95% CI: 0.45–0.95 non-HRRm: 42.1 vs. 38.9; HR: 0.89, 95% CI: 0.70–1.14 BRCAm: NR vs. 23.0; HR: 0.29, 95% CI: 0.14–0.56 non-BRCAm: 39.6 vs. 38.0; HR: 0.91, 95% CI: 0.73–1.13
TALAPRO-2 (Talazoparib/placebo + enzalutamide)	1L mCRPC	HRRm (20%)/non-HRRm BRCA1m (5%) BRCA2m (23%)	24 m	ITT: not reached vs. 21.9; HR: 0.63, 95% CI: 0.51–0.78; *P* < 0.001 HHRm: 27.9 vs. 16.4; HR: 0.46, 95% CI: 0.30–0.70, *P* < 0.001 non-HRRm: not reached vs. 22.5; HR: 0.70, 95% CI: 0.54–0.89; *P = 0.009	ITT: HR: 0.89; *P* = 0.35 (no mature data—only 31% of events)
MAGNITUDE (IA2) Niraparib/Placebo + Abiraterone and prednisone	1L mCRPC	HRRm (100%) BRCAm (53.2%)	35 m	HHRm: 16.7 vs. 13.7; HR: 0.76, 95% CI: 0.60–0.97 BRCAm: 19.5 vs. 10.9; HR: 0.55, 95% CI: 0.39–0.78, *P* = 0.0007	HHRm: 29.3 vs. 32.2; HR: 1.01, 95% CI: 0.75–1.36 BRCAm: 30.4 vs. 28.6; HR: 0.788; 95% CI: 0.554–1.120; *P* = 0.1828

PARPi: poly (ADP-ribose) polymerase inhibitor; mCRPC: metastatic castration resistant prostate cancer; HRR: homologous recombination repair; HRRm: HRR mutated; BRCA: breast cancer; rPFS: radiological progression-free survival; OS: overall survival; ITT: intention to treatment; BRCAm: BRCA mutated; FU: follow-up; * same BRCAm rates in the two treatment groups; NR: not reported

To date, we are awaiting the publication of data from two phase 3 studies: the CASPAR (NCT04455750) and the AMPLITUDE (NCT04497844) trials, which investigated the combination of enzalutamide plus rucaparib versus enzalutamide alone and niraparib plus abiraterone versus abiraterone alone, respectively [45, 46]. Both studies enrolled a population non-selected for HRR mutations.

## Discussion

Patients with mCRPC have a median survival of about 3 years in clinical trials (which is reduced to 2 years in clinical practice). A significant proportion of these patients receive only one line of therapy, which therefore must be carefully selected [47]. Patients with *BRCA* alterations, especially *BRCA2*, had significantly worse median PFS and OS, compared to BRCA wild type cases (3.7 months vs. 9.9 months, *P* < 0.001; 18.9 months vs. 33.9 months, *P* < 0.001, respectively) [8].

Given the poor prognosis in these patients, the choice of first-line treatment becomes increasingly important. In fact, until recently, the first line treatment strategy of BRCA mutated mCRPC was represented by an ARSi or chemotherapy plus ADT, while PARPi was intended for patients with *BRCA* mutation after a previous line with an ARSi, based on the results of the PROfound trial [39, 40].

There are to date three different large, randomized trials (TALAPRO-2, PROpel, MAGNITUDE) that investigated the same therapeutic strategy (combination of PARPi + ARSi) in first line treatment for mCRPC patients. These studies aimed to expand the population of mCRPC patients who may benefit from PARPi, even in an earlier phase of disease.

The PROpel study was a formally positive study, as it demonstrated a statistically significant increased rPFS in the all-comers population (not selected for HRR mutational status) treated with abiraterone + olaparib, as compared with abiraterone alone. However, the final pre-specified survival analysis, at a median follow-up of 36.6 months (47.9% of events), did not demonstrate a clear OS advantage of the combination therapy in the HRR non-selected population. Interestingly, post-hoc mutational analysis of tumor samples and ctDNA helped to select patients who benefit most from the addition of PARPi to ARSi, and this is true both in the subgroup of patients with *HRR* gene mutations, and even more in tumors with *BRCA* gene mutations (where olaparib + abiraterone lead to a 71% reduction in the risk of death; HR: 0.29). It should be noted, however, that these sub-group analyses were conducted in a limited cohort of patients [24]. This evidence supported an earlier use of the PARPi in combination with an ARSi in clinical practice, moreover in mutated HRR patients. Based on these results, EMA approved the combination of abiraterone and olaparib in advanced CRPC patients not susceptible to chemotherapy, regardless of HRR mutational status [42].

The TALAPRO-2 study showed that the combination of talazoparib + enzalutamide versus placebo + enzalutamide was able to significantly prolong the rPFS of mCRPC unselected for the mutational status of HRR genes, although the extent of the benefit is greater in the HRR-deficient population. So far, OS data of the study are still immature (only 31% of events, after 24 months of follow-up) [26]. In addition, the use of this combination is affected by significant hematological toxicity. Therefore, the increased toxicity related to this combination could represent a limitation in the use in clinical practice, in favor of the other combinations that gave overlapping toxicity to the control arm.

The MAGNITUDE study was a formally positive study, confirming the statistically significant rPFS advantage of the combination of niraparib + abiraterone over placebo + abiraterone in a population selected for mutational status (*BRCA* mutated) with mCRPC in first line treatment. However, the rPFS outcome did not translate into a clear prolongation of OS, and in a *BRCA* mutated population, this is discouraging. However, a longer follow-up is needed in order to have more maturity survival data [25, 44].

These trials, that investigated the role of the ARSi and PARPi combination, are heterogeneous for the study design and enrolled populations. For this reason, we cannot compare these studies directly. Despite the heterogeneity of these trials, all three of those involve two questions that need to be answered. The first question is if there is a synergy between PARPis and ARSis, permitting their combination to improve the clinical efficacy of ARSi in first line setting of mCRPC, regardless of mutational status. The second question is if the rPFS can be considered an OS surrogate endpoint. The results are inconclusive because a clear clinical benefit was not achieved and the data of rPFS and OS were divergent, especially in “all comer” patients. In our opinion, more mature follow-up data are needed and new phase III randomized studies are awaited to solve these issues.

However, a clear message from these trials is that the mutational status has an important role on the efficacy of combination therapy because it has been shown it conditions the entity of the benefit in the various sub-populations analyzed (*BRCA* mutated > HRR-deficient > all-comers > HRR-proficient). The non-concordant results, derived from these studies, may be explained by the different agents (and their dose) used, and the different populations enrolled.

PARPis used in TALAPRO-2, MAGNITUDE and PROpel have different properties and ability to inhibit PARP. While olaparib (PROpel) hinders PARP-1, 2, and 3, niraparib (MAGNITUDE), as well as talazoparib (TALAPRO-2) act against PARP-1 and 2 only. Moreover, full dose olaparib was administered in PROpel, while a reduced dose of niraparib (200 mg instead of 400 mg) and of talazoparib (0.5 mg instead of 1 mg) were used in MAGNITUDE and TALAPRO-2, respectively. Similarly, abiraterone and enzalutamide have different mechanisms of action. Abiraterone is an androgen synthesis inhibitor, while enzalutamide is an AR inhibitor and this difference may support an inconsistent synergy with PARPi.

In relation to the study population, PROpel and TALAPRO-2 have randomized unselected mCRPC populations, whereas MAGNITUDE pre-screened eligible patients for mutational status, but mainly there were differences on previous treatments permitted to enrolled patients. In fact, antecedent abiraterone administration was not authorized in PROpel, whereas allowed in MAGNITUDE, if given for less than 4 months in the first line mCRPC setting and in TALAPRO-2, if administered in the hormone-sensitive state. Previous androgen-receptor inhibitor use was not allowed in TALAPRO-2, but was permitted in PROpel, if therapy finished more than 12 months prior to enrollment. ARSi use was authorized also in MAGNITUDE, if administered in the hormone-sensitive metastatic PCa or in non-metastatic castrate-resistant states.

A recent meta-analysis of all three trials demonstrated that the first-line combination of PARPi + ARSi is associated with a nearly 35% improvement in rPFS in all comer mCRPC patients (HR: 0.65). A statistically significant rPFS benefit was observed in the non-HRRm cohort (HR: 0.74), although, as anticipated, the magnitude of effect was larger in HRRm (HR: 0.55) [48]. In the overall cohort, pooled OS analysis from PROpel and TALAPRO-2 demonstrated a 16% OS improvement (HR: 0.84), while in HRRm cohort (HR: 0.79) and in the *BRCA1/2* mutated cohort (HR: 0.53) the effect was larger but not statistically significant. With regards to safety, unfortunately, the relative risk of high-grade treatment emergent anemia was increased by approximately six-fold, suggesting a limited use of combination therapy [48]. The results of this interesting meta-analysis resume how far we are from observing a reliable clinical advantage of combination therapy, as compared with the already approved first line treatment, represented by ARSi alone in mCRPC patients.

Focusing on sequential administration of these agents, in our opinion, study designs of these three trials lack functional crossover, beyond the first line treatment. In standard practice in 2024, if a patient progresses on abiraterone or enzalutamide, PARPis would be considered based on an HRR mutation. However, in MAGNITUDE trial, only around 30% of patients on the control arm with a mutation, benefitting from a PARPi, received subsequent PARPi therapy. At the same time, also in TALAPRO-2 and PROpel trials, a low percentage of patients with this characteristic had PARPi, confounding the true impact of these treatments.

In this regard, recently a phase III radomized study (TRITON3) demonstrated that after an ARSi-based first line therapy, rucaparib significantly prolonged rPFS in tated mCRPC population, compared to a treatment chosen by the investigator (ARSi or docetaxel), reducing the risk of progression. However, the limitations of a short follow-up and high crossover rates prevented rucaparib to show a significant advantage in OS. So the TRITON3 trial could suggest a potential therapeutic sequence strategy for BRCA mutated patients, indicating the major benefit from the PARPi second line treatment after an ARSi, with respect to docetaxel or another ARSi [36].

In summary, the combination of abiraterone and olaparib (PROpel trial) [24] demonstrated an increased median PFS (mPFS), with OS data not yet mature, in a population non-selected for HRR status. This study changed our clinical practice and leads to approve this combination in patients not eligible for chemotherapy, regardless of HRR status. The combination of talazoparib and enzalutamide (TALAPRO-2 trial) [26] was superior in terms of mPFS as opposed to ARSi monotherapy in HRR non-selected population, but with important hematological toxicity, which makes this combination difficult to apply in clinical practice. The combination of niraparib and abiraterone (MAGNITUDE trial) [25] was demonstrated to be superior to abiraterone monotherapy in patients with *BRCA* mutation, but not in HRR-proficient patients. Despite that, in the mutated *BRCA/HRR* deficient population we do not know what is the added value of the combination of PARPi + ARSi as compared with a PARPi monotherapy. Then, it would be interesting to study this aspect with a clinical trial, comparing the above combination with PARPi monotherapy in the *BRCA* mutated population.

## Conclusions

In mCRPC setting, it has been shown that anticipating the use of PARPi in combination with an ARSi, regardless of patient HRR state, seems to be more beneficial in terms of rPFS, supporting the synergism of these two different drugs. Therefore, olaparib in combination with abiraterone may represent an important therapeutic option, especially in patients not eligible for chemotherapy treatment. In our opinion, when weighing toxicity against survival, up to now, the combination therapy has not guaranteed a clear clinical benefit, if we compare it with a sequencing regimen, in all-comers population.

Speaking of mutated *BRCA* patients, it has been demonstrated that PARPis are superior to ARSi or docetaxel chemotherapy in the castration resistant setting, but we do not know the real advantage of a combination with an ARSi as compared with PARPi alone. Further trials need to be clarified these concerns.

## Abbreviations

ADT: Androgen Deprivation Therapy
AEs: adverse events
AR: androgen receptor
ARSi: androgen receptor signalling inhibitor
BRCA1: breast cancer 1
CRPC: castration resistant prostate cancer
DSB: double-strand breaks
GC: gene conversion
HRR: homologous recombination repair
HRRm: homologous recombination repair-mutated
mCRPC: metastatic castration resistant prostate cancer
MMEJ: microhomology-mediated end joining
NHEJ: non-homologous end joining
OS: overall survival
PARP: poly (ADP-ribose) polymerase
PARPi: poly (ADP-ribose) polymerase inhibitor
PCa: prostate cancer
PFS: progression-free survival
rPFS: radiological progression-free survival
SSA: single strand annealing
SSBs: single strand breaks

## Declarations

### Copyright

© The Author(s) 2024.
